# Virus-Induced NETs – Critical Component of Host Defense or Pathogenic Mediator?

**DOI:** 10.1371/journal.ppat.1004546

**Published:** 2015-01-08

**Authors:** Craig N. Jenne, Paul Kubes

**Affiliations:** 1 Department of Microbiology, Immunology and Infectious Diseases, Calvin, Phoebe & Joan Snyder Institute for Chronic Diseases, University of Calgary, Calgary, Alberta, Canada; 2 Department of Physiology and Pharmacology, Calvin, Phoebe & Joan Snyder Institute for Chronic Diseases, University of Calgary, Calgary, Alberta, Canada; University of Michigan Medical School, United States of America

## Viruses and Neutrophils: An Unlikely Pair

Much of the effort to understand and regulate the host antiviral response has been focused on adaptive immunity including high affinity, neutralizing antibody production, and the generation of specific “killer” CD8+ T cells. Current vaccine approaches target these effector pathways, and antibody titers or numbers of antigen-specific lymphocytes are often used as measures of the immune responses. Although these responses are dominated by lymphocytes, the innate immune system also plays a central role in the host antiviral response. Innate immunity is essential for antigen presentation and cytokine production, helping to direct and shape the ensuing adaptive response. Also of note is the observation that most viral infections elicit a robust, multifaceted inflammatory response.

If we stop and consider the bigger picture, the presence of an inflammatory response should not be all that surprising. Innate immunity, and by extension inflammation, is often our body's first line of defense, functioning to isolate and limit infection. Furthermore, the innate immune system expresses several pattern recognition receptors (PRR) specific for viral ligands (TLR3, 7, 8, RIG-I, MDA5) and produces a number of potent antiviral mediators (IFN, TNF-α, IL-15, IL-18) [1]–[3], illustrating that innate immunity has evolved to directly deal with viruses. Although neutrophils are central players in this acute inflammatory response and are rapidly recruited to sites of viral infection (often comprising more than 70% of the leukocyte infiltrate), their specific role in host viral immunity remains somewhat confusing. In some studies, using highly pathogenic strains of influenza, neutrophils have been demonstrated to be critical in limiting viral replication and disease progression during the early phases of infection [4], [5], whereas others have reported that neutrophil recruitment to the lung in response to viral infection is associated with increased epithelial cell death, fibrin deposition, and a worse prognosis [3], [6], [7]. Some of the confusion regarding the role neutrophils play in viral infection can be attributed to an incomplete understanding regarding the effector mechanisms neutrophils use to deal with viruses.

## NETs: What Are They?

Much of the work studying the role of neutrophils in viral infection has focused on classic effector mechanisms (reactive oxygen species, degranulation, cytokine production). Nearly ten years ago, a “new” effector mechanism was described for the first time, neutrophil extracellular traps (NETs) [8]. NETs are structures comprised of a sticky, complex mesh of decondensed strands of nuclear DNA released into the extracellular environment. These chromatin webs carry a strong negative charge and are studded with both nuclear proteins, such as histones (comprising up to 70% of NET proteins), and proteins derived from the neutrophil granules, including defensins, elastase, cathepsins, lactoferrin, and myeloperoxidase (MPO) [8], [9].

NET formation and release occurs following decondensation of nuclear DNA in response to a number of different stimuli and appears to involve activity of MPO, neutrophil elastase, and peptidylarginine deiminase type IV (PAD4), since inhibition or deficiency in any of these enzymes negatively affects NET production [10]–[12]. Depending on the location of the neutrophil when stimulated (extravasated versus vascular), these NETs can be either spread throughout the interstitium of specific organs or released into the lumen of blood vessels, where they may attach to the vessel wall of narrow capillaries. Once deployed, NETs act to ensnare and kill passing pathogens. These structures are extremely effective at limiting bacterial dissemination from a site of infection, and they act to “filter” the blood of circulating pathogens. NETs in many ways are an equalizer, allowing the relatively slow moving cells of the immune system to “catch” highly motile or circulating bacteria, basically turning neutrophils into spider-like predators; setting traps and waiting for the prey to come to them. Disruption of the structure of NETs through treatment with DNAse results in pathogen “escape” and bacterial dissemination demonstrating exactly how important this effector mechanism is in controlling bacterial infections [13].

## Virally-Induced Nets

Although NETs were first identified almost a decade ago, only now are we beginning to understand what role this unique neutrophil effector mechanism plays in viral immunity. Recently it has become clear that viral infection, or more specifically, virally derived molecules, many of which act as pathogen-associated molecular patterns (PAMPs), are potent inducers of NET production. To date, no single specific signal has been identified as responsible for NET release, but rather this effector response has been attributed to a wide variety of stimuli including TLR-ligands, phorbol esters, complement, and the binding of activated platelets to neutrophils. Moreover, a number of viruses induce NET formation, including influenza A, HIV-1, myxoma and encephalomyocarditis virus [14]–[17].

Interestingly, these viruses do not infect the neutrophil per se, but rather are either detected by recognition of viral particles by pattern recognition receptors PRR on the neutrophil (TLR7, TLR8) or via secondary signals produced upon infection of other host cells (epithelium, Kupffer cells, platelets) [15]–[17]. The use of secondary signals to induce NET production has important advantages in the context of viral infection. First, the ability to respond to a fixed array of host-derived molecules produced by virally infected cells allows the neutrophil to “recognize” a diverse spectrum of viruses that frequently and rapidly alter their surface antigens. Second, whereas many of the bacteria that trigger NET release are extracellular pathogens and are easily directly detected by the neutrophils, viruses result in intracellular infections and as such can “hide” from the neutrophil. By responding to secondary signals released from infected cells, the neutrophil is able to efficiently detect intracellular infections and to participate in host antiviral immunity.

### NETing Viruses

**Figure 1:**
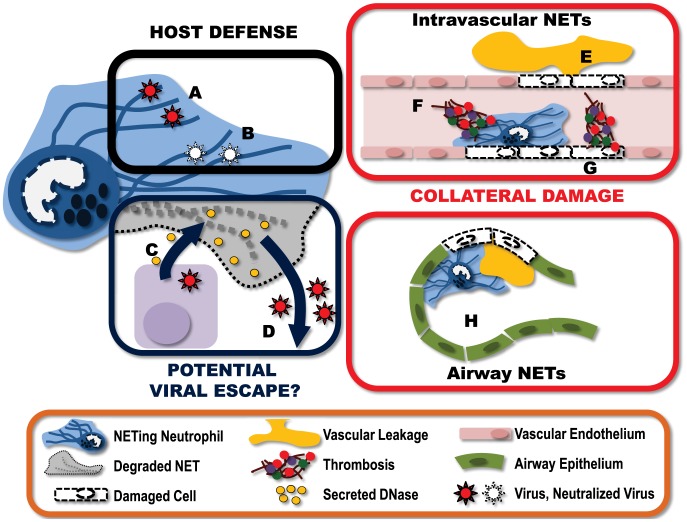
Virally-induced NETs represent a complex and multifaceted component of the host immune response. **A**) NETs can “catch” virus particles, preventing the virus from reaching target cells. **B**) These ensnared viruses can then be neutralized by NET-associated host proteins such as MPO and defensins. To counter this, some viruses have developed countermeasures. **C**) Some viruses express endonucleases, which are produced and released by infected host cells. These endonucleases have the potential to break down nearby NETs. **D**) Destruction of the NET structures may help ensure the “escape” of virions released from the infected cell. This immune response does not come without a price, however. **E**) NETs produced within the vasculature damage endothelium, resulting in vascular leakage of plasma into the extravascular space. **F**) Damage to endothelium exposes the subendothelium, triggering the binding of platelets and the activation of thrombin leading to the formation of intravascular thrombi. **G**) Additionally, NET-associated proteins can also directly activate coagulation, further amplifying the generation of intravascular thrombi. **H**) NETs formed within the alveolar spaces of the lung can obstruct airflow and reduce gas exchange. **I**) Furthermore, NETs within the airways damage the epithelium, leading to fluid accumulation within the airspace, further impeding lung function.

The recognition that viruses are able to induce NET formation has initiated new research efforts to understand how this neutrophil effector mechanism contributes to the host antiviral response. To date, two different mechanisms have been identified by which NETs can protect the host from virus infection; sequestration and neutralization (Fig. 1). Studies both in vitro and in vivo have demonstrated that the sticky, web-like structure of NETs can bind and sequester virions, preventing them from reaching their target cells [15], [16]. In these studies the ability to bind virions was directly attributable to the DNA structure of the NET and was compromised following treatment of NETs with DNAse. Paradoxically, although bacterially-induced NETs (LPS) are able bind and neutralize viruses, virally induced NETs appear to have limited capacity to clear bacterial infections suggesting there may be a fundamental difference in the specific nature or NETs induced by either bacteria or viruses [16], [18], [19].

In addition to viral capture, NETs have also been shown to directly neutralize the viral particles. This virus neutralization involves MPO and defensins, which are granule-derived proteins associated with NETs, and goes beyond simply sequestering the virus in the NET since virions recovered from the NET have reduced capacity to infect target cells [15]. Treatment with either an MPO inhibitor or antidefensin antibodies reduced the capacity of NETs to neutralize HIV-1 virions. Furthermore, the NET protein α-defensin directly inhibits influenza replication and protein synthesis [20], [21]. Moreover, viral fusion is blocked when viruses are bound by molecules carrying a strong negative electrostatic charge such as those present on the DNA strands of NETs [22]. Thus NETs represent a multilayered defense against viral infection, sequestering viral particles, preventing fusion of virus with target cells and direct neutralization of virions.

Interestingly, a number of viruses, for example those from the herpesvirus family, express viral proteins with endonuclease activity [23]. Although these enzymes are critical in viral genome processing, it is possible these viral products may confer a previously unrecognized advantage to the pathogen. Expressed late in the viral replication cycle, these nucleases have the potential to be released upon virus-induced lysis of the host cell into the extracellular environment, where they can degrade NETs and enhance viral escape or dissemination (Fig. 1).

## Protection: But at What Price?

As with most host immunity, NET-mediated protection from pathogens comes at some cost to the host (Fig. 1). NETs are extremely cytotoxic. These sticky structures are covered with molecules designed to kill pathogens and, unfortunately, fail to differentiate between friend and foe. Many of the individual NET components, including elastase and histones, are cytotoxic, leading to endothelial damage, exposure of the subendothelium, coagulation, and exacerbated inflammation [24]–[27]. The very nature of the structure of NETs further enhances their cytotoxicity. NETs prevent the diffusion of neutrophil granular proteins within the extracellular environment, instead concentrating the antimicrobial (cytotoxic) molecules, and through the “stickiness” of the DNA strands, adhering this potentially damaging structure to the surface of host cells. The end result is significant potential for collateral damage.

In a model of systemic bacterial infection, the production of NETs is directly responsible for extensive liver damage; a pathology that is completely preventable with i.v. administration of DNAse to degrade the NET structures [13]. Within the context of viral infection, excessive neutrophil and NET deposition has been reported in severe influenza infections. Immunohistochemical analysis of lungs from infected mice demonstrates pronounced staining for NETs (DNA, histone, and MMP9) within both the blood vessels and the airspaces [14]. The presence of NETs in this mouse model of influenza infection appears to be associated with increased alveolar capillary damage, hemorrhagic lesions, and obstruction of the small airways.

It is important to note that this “collateral damage” may not be all bad. Although NETs deposited on host cells can inflict significant damage, resulting in cell death, there may be some benefit to this area-effect of NETs. If NETs are released in close proximity to infected host cells, this collateral damage may actually serve to kill virally infected cells and thereby limit the propagation and spread of the virus. In this way, NETs may have a dual function in viral immune responses, actively eliminating infected cells and catching virus released from the cells they fail to kill.

Many questions remain including the actual proteolytic activity of NETs in plasma, where significant antiproteases dominate, the mechanism by which NETs adhere to intravascular as well as extravascular structures, and the persistence of NETs in the presence of endogenous host DNAses. Although we are only beginning to understand the role of NETs in viral infection, it is clear that they have the capacity to both protect and damage the host. Further investigation into the interactions between NETs and viruses is needed to identify new immunomodulation strategies to enhance antiviral host responses while simultaneously limiting collateral host tissue damage.
